# In Endometrial Cancers Originating from the Uterine Corpus, Does the Location of the Tumor Affect the Prognosis?

**DOI:** 10.3390/jcm14186484

**Published:** 2025-09-15

**Authors:** Gökşen Görgülü, Emel Doğan Özdaş, Erol Özdaş, Özge Küçükatalay, Esin Kasap, Tuğba Karadeniz, Muzaffer Sancı

**Affiliations:** 1 Department of Gynecologic Oncology, University of Health Sciences, İzmir Tepecik Training and Research Hospital, Sanayi Street No:7, 35020 Bornova, Izmir, Turkey; egecim2704@gmail.com (E.D.Ö.); emelege06@hotmail.com (E.Ö.); drsanci@yahoo.com (M.S.); 2 Department of Gynecology and Obstetrics, University of Health Sciences, İzmir Tepecik Training and Research Hospital, Sanayi Street No:7, 35020 Bornova, Izmir, Turkey; ozgekucukatalay@gmail.com (Ö.K.); dresincelik@windowslive.com (E.K.); 3 Department of Pathology, University of Health Sciences, İzmir Tepecik Training and Research Hospital, Sanayi Street No:7, 35020 Bornova, Izmir, Turkey; tugbakaradeniz@gmail.com

**Keywords:** endometrioid adenocarcinoma, uterine corpus, endometrial cancer, uterine wall

## Abstract

**Objectives:** This study examines the influence of the uterine wall region from which a tumor originates on the prognosis of endometrial cancers (ECs) originating from the uterine corpus without lower uterine segment involvement (LUSI). **Methods:** This study included 68 EC patients who were operated on between January 2015 and January 2022 and met the following criteria: no LUSI noted in the final pathology report; endometrioid adenocarcinoma originating from the uterine corpus, as noted in the histopathology report; and Stages 1A and 1B tumors, classified according to the FIGO 2009 staging system. From the final pathology results after the operation, these patients were divided into four groups according to the uterine wall region from which the tumor originated: the anterior wall, posterior wall, fundus, and lateral wall. **Results:** No statistically significant difference was recorded between the groups regarding age, BMI, comorbidity, nulliparity, stage, grade, tumor size, DFS, and OS (*p* > 0.05). LVSI was detected at a higher rate in the anterior and posterior wall groups than in the fundus and lateral wall groups (*p* = 0.038). Similarly, the rates of recurrence and death were statistically notably higher in the anterior and posterior wall groups in comparison with those in the other two groups (*p* < 0.001). **Conclusions:** In endometrioid adenocarcinomas of the uterine corpus, tumors originating from the anterior and posterior walls have a worse prognosis than those originating from the fundus and lateral wall. Closer follow-up may be necessary for endometrial cancers originating from the anterior and posterior uterine walls.

## 1. Introduction

Endometrial cancer (EC) is a uterine malignancy with globally increasing mortality and incidence rates [1]. It is the sixth most frequently diagnosed cancer in women [2] and is most prevalent between the ages of 65 and 75. Geographical differences, socioeconomic status, and race are determinants of EC mortality and incidence rates [3,4].

EC is often identified in its initial stages, which is a promising indicator of prognosis [5]. Prognostic factors for endometrial cancer include factors other than the stage, such as the histological grade, tumor volume, presence of LVSI (lymphovascular space invasion), myometrial invasion depth, and lymph node involvement [6]. Lower uterine segment involvement (LUSI) originating from the UC is a poor prognostic factor in EC, and tumors originating from direct LUSI have been reported as a poor prognostic factor [7,8].

Anatomical variations in the uterine wall thickness and lymphovascular drainage pathways may influence tumor behavior within the corpus. Fundus and upper corpus regions typically drain to para-aortic lymph nodes, whereas lower uterine segments and lateral walls drain to pelvic iliac nodal basins, potentially altering patterns of metastasis and recurrence [9]. Furthermore, myometrial thickness varies based on location within the corpus: anterior and posterior walls are generally thicker than the fundus, and both are significantly thicker than the isthmus or cornual regions [10]. These anatomical differences may partially explain the prognostic differences observed according to tumor location.

EC is classified according to its site of origin in two sections of the uterus, the LUS (lower uterine segment) and the UC (uterine corpus), with tumors of UC origin being more common [11,12]. As of yet, no study in the literature has examined the uterine wall from which the tumor develops in EC patients. We hypothesize that just as tumors originating from the LUS are associated with a poor prognosis, the location of involvement may affect the prognosis in tumors originating from the UC. In this study, our goal was to evaluate the correlation between the uterine wall region in which the tumor originates and the prognosis of endometrial cancers originating from the non-LUSI uterine corpus.

## 2. Materials and Methods

### 2.1. Study Design and Patient Selection

This retrospective cohort study was conducted at the University of Health Sciences, İzmir Tepecik Training and Research Hospital. Institutional ethical approval was obtained prior to data collection (approval No: 2022/02-13, date: 15 February 2022). All procedures conformed to the ethical standards outlined in the Declaration of Helsinki and the Good Clinical Practice guidelines.

**Figure 1:**
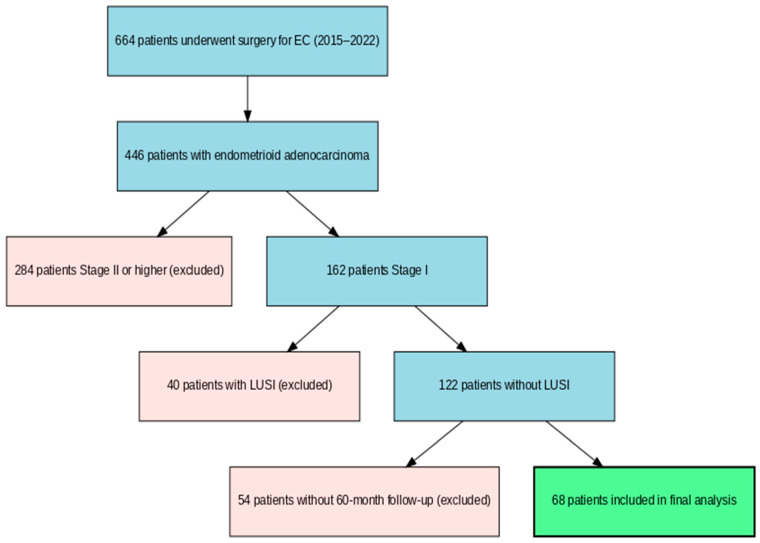
Patient flow diagram.

Between January 2015 and January 2022, 664 patients underwent surgery for endometrial cancer at our clinic. Of these patients, 446 had endometrioid adenocarcinoma, 284 of which were Stage 2 or higher. Of the remaining 162 patients, 40 had LUSI. Furthermore, of the remaining 122 patients, 54 did not have a 60-month follow-up period (Figure 1). A total of 68 patients who underwent primary surgical treatment for endometrial cancer between January 2015 and January 2022 were included. The inclusion criteria were as follows: (1) a diagnosis of endometrioid-type endometrial adenocarcinoma, (2) tumors originating from the uterine corpus without lower uterine segment involvement (LUSI), (3) FIGO 2009 Stage 1A or 1B disease, (4) a follow-up period of at least 60 months, and (5) complete medical and pathological records available. The exclusion criteria included non-endometrioid or mixed histology, the presence of LUSI, Stage II or higher disease, or loss to follow-up.

### 2.2. Surgical Procedure

All patients underwent total abdominal hysterectomy with bilateral salpingo-oophorectomy. An intraoperative frozen section analysis was performed to assess the depth of myometrial invasion and tumor grade, using a cryostat (CryoStar NX50, Thermo Fisher Scientific, Waltham, MA, USA). Patients with ≥50% myometrial invasion or high-grade tumors (Grade 3) underwent pelvic and para-aortic lymphadenectomy. The surgeries were performed by a consistent team of gynecologic oncologists using standardized techniques.

### 2.3. Pathology Evaluation

Tumor location within the uterine wall was determined based on postoperative final pathology reports, using both macroscopic gross examination of the surgical specimen and microscopic histopathological evaluation. In cases where the macroscopic and microscopic findings differed, the final classification was based on the microscopic assessment. All pathology evaluations were performed by two independent gynecologic pathologists blinded to the clinical outcomes. The inter-rater reliability was assessed based on Cohen’s kappa coefficient, and disagreements were resolved via consensus review. Histopathological evaluation was performed using hematoxylin and eosin (H&E) staining (Dako, Agilent Technologies, Glostrup, Denmark). Postoperative pathology reports were reviewed to confirm the histological subtype, tumor grade, tumor size, depth of myometrial invasion, lymphovascular space invasion (LVSI), and lymph node status. The tumor origin was classified based on the region of the uterine wall in which the tumor was predominantly located, as described in the final pathology report and confirmed by the pathologist. The patients were categorized into four groups based on tumor location: anterior wall, posterior wall, fundus, and lateral wall.

### 2.4. Adjuvant Therapy

Adjuvant therapy indications and protocols followed the National Comprehensive Cancer Network (NCCN) guidelines in effect during the study period. All patients who met the criteria for adjuvant treatment received standardized regimens for their respective risk groups, including external beam radiotherapy, vaginal brachytherapy, or chemotherapy, as indicated. Treatment protocols and dosages were consistent across all tumor location groups to minimize treatment-related variability that could confound survival analysis. No adjuvant treatment was administered to patients with Grades 1–2 tumors, <50% myometrial invasion, and LVSI negativity. Vaginal brachytherapy was given to patients with ≥50% myometrial invasion but negative LVSI and to those with Grades 1–2 tumors and positive LVSI. Patients with Grade 3 tumors and/or ≥50% myometrial invasion received external beam radiotherapy (EBRT), provided they had negative lymph nodes after surgical staging.

### 2.5. Follow-Up Protocol

Patients were followed at 3-month intervals during the first two years, every 6 months for the next three years, and annually thereafter. Clinical evaluation and imaging (MRI or CT scan) were performed at each visit to assess for recurrence. Magnetic resonance imaging (MRI) examinations were performed using a 1.5-Tesla scanner (Siemens Healthineers, Erlangen, Germany), while computed tomography (CT) scans were acquired with a 64-slice scanner (Toshiba Medical Systems, Tokyo, Japan). Disease-free survival (DFS) was defined as the time from primary surgery to either recurrence or last follow-up. Overall survival (OS) was calculated from the date of surgery to death or last follow-up.

### 2.6. Statistical Analysis

SPSS (SPSS Statistics version 22.0, IBM Corp., Armonk, NY, USA, SPSS Inc.) statistical software was used for statistical analysis. Statistical comparisons were made using Scheffe’s post hoc test in the analysis of variance, the Kruskal–Wallis tests for nonparametric variables, and ANOVA for parametric values. To assess categorical data, both Fisher’s exact tests and the Pearson chi-square test were utilized. Categorical variables were presented as numbers and percentages (*n*; %), and numerical variables were presented as median [quartile] values. A *p* value of <0.05 was considered statistically significant. Disease-free survival (DFS) and overall survival (OS) were estimated using the Kaplan–Meier method. Differences between survival curves were assessed with the log-rank test. Survival time was calculated from the date of surgery to the date of recurrence, death, or last follow-up. Patients without events at the end of follow-up were censored at their last known disease-free or alive date. A *p* value of <0.05 was considered statistically significant.

## 3. Results

Over the course of this study, 664 patients diagnosed with EC underwent surgery, and 68 of those who met the specified criteria were incorporated into this study. The mean age of all patients was 58.5 (54–67.75) years. The mean BMI was 36 (30.8–43.8) kg/m^2^. Diabetes mellitus (DM) was present in 15 (22.1%) patients, and hypertension was present in 19 (27.9%) patients. Only one (1.5%) patient was nulliparous. According to the postoperative pathology results, tumors were observed on the anterior wall in 13 (19.1%) patients, on the posterior wall in 10 (14.7%) patients, on the fundus in 41 (60.3%) patients, and on the lateral wall in 4 (5.9%) patients. When all patients were analyzed regarding the grade, 17 (25%) patients had Grade 1 tumors, 47 (69.1%) patients had Grade 2 tumors, and 4 (5.9%) patients had Grade 3 tumors. The mean tumor diameter was 40 (30–50) mm. According to the FIGO 2009 staging system, 51 (75%) patients had Stage 1A EC, and 17 (25%) patients had Stage 1B EC. A total of 16 (23.5%) patients had LVSI, and 8 (11.8%) patients had recurrence. During follow-up, eight (11.8%) patients experienced no recurrence (ex). The demographics, survival results, and final postoperative pathology findings of all patients are presented in Table 1.

**Figure 2:**
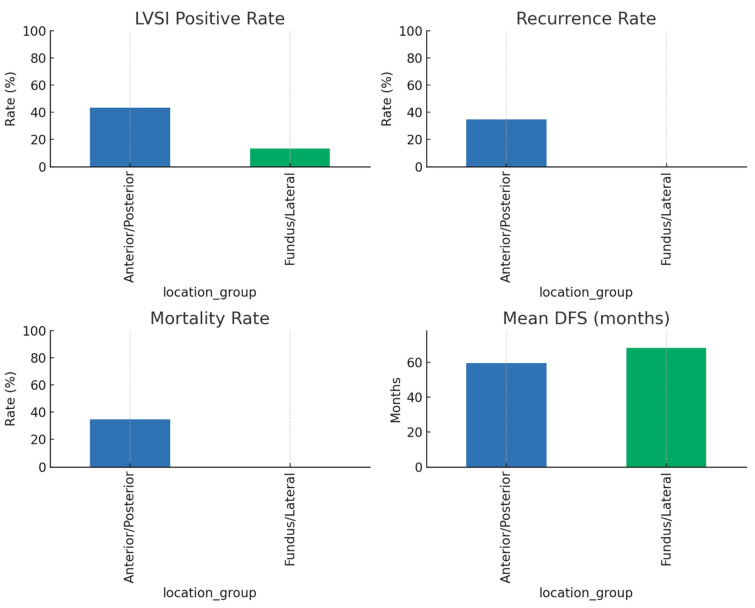
Oncologic outcomes according to tumor location in the uterine corpus.

The patients were separated into four groups according to the region of the uterine wall in which the tumor originated: the anterior wall, posterior wall, fundus, and lateral wall. The mean age of patients in the anterior group was 58 (53–67) years, and their BMI was 37.5 (36–44) kg/m^2^; the mean age of patients in the posterior group was 60 (57–63) years, and their BMI was 34.2 (30–44) kg/m^2^; the mean age of patients in the fundus group was 56 (52–63) years, and their BMI was 33 (27–44) kg/m^2^; and the mean age of patients in the lateral group was 64 (57–68) years, and their BMI was 36 (36–36) kg/m^2^ (*p* = 0.388, *p* = 0.520, Table 2). When these four groups were analyzed in terms of the presence of diabetes mellitus and hypertension, no significant difference was found between the groups (*p* = 0.497, *p* = 0.616, Table 2). When demographic characteristics (age, BMI, parity, and systemic disease) were compared between these four groups, no meaningful difference was observed (*p* > 0.05, Table 2). When the groups were compared according to the features in the final postoperative pathology report, no statistically significant differences were discovered in regard to grade, stage, and tumor size (*p* = 0.661, *p* = 0.501, *p* = 0.320). LVSI was detected at a statistically higher rate in the anterior and posterior groups than in the fundus and lateral groups (*p* = 0.038, Table 2, Figure 2).

**Figure 3:**
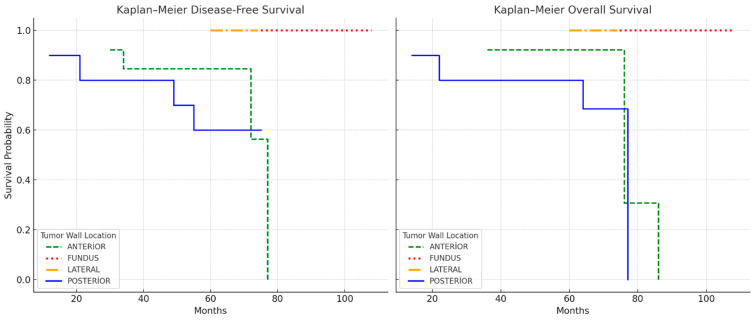
DFS and OS Kaplan–Meier curves based on tumor wall location.

When it came to comparing survival between the groups, no meaningful difference was observed according to DFS and OS (*p* = 0.310, *p* = 0.632), while higher recurrence and mortality rates were observed in the anterior and posterior groups within the span of the 60-month follow-up period (*p* < 0.001, Table 2, Figure 2). Although recurrence and mortality were more frequent in anterior/posterior tumors, these differences did not translate into statistically significant differences in DFS or OS in the Kaplan–Meier analysis (Figure 3), likely due to the limited number of events.

An odds ratio analysis revealed that LVSI was present in 43.5% of anterior/posterior tumors compared with 13.3% of fundal/lateral tumors (OR 5.00, 95% CI 1.52–16.45, *p* = 0.013). Recurrence was observed in 34.8% of anterior/posterior tumors, whereas no recurrence occurred in fundal/lateral tumors (OR 48.00, 95% CI 2.60–886.66, *p* < 0.0001). Similarly, mortality was 34.8% in anterior/posterior tumors and absent in fundal/lateral tumors (OR 48.00, 95% CI 2.60–886.66, *p* < 0.0001). These findings indicate that a tumor located on the anterior or posterior wall is associated with significantly higher LVSI and worse oncologic outcomes (Table 3).

## 4. Discussion

This study found that the presence of LVSI and the ex rate were higher in EC tumors originating from the anterior and posterior walls of the UC compared with tumors originating from the fundus and lateral walls.

Increased BMI (obesity), postmenopausal status, advanced age, nulliparity, and systemic diseases such as DM and HT are known to be important demographic risk factors for the development of EC [13]. In a study conducted by Phelan et al., endometrial cancer patients with and without lower uterine segment involvement were compared in terms of age, nulliparity, hypertension, and the presence of diabetes mellitus, and no statistically meaningful difference was observed [14]. In our study, tumors originating from the uterine corpus were categorized into four different regions, and no notable differences were detected regarding age, BMI, hypertension, or the presence of diabetes mellitus.

In a study conducted by Kizer et al. which included 481 patients, no meaningful difference was found between corpus (*n* = 258) and lower uterine segment (*n* = 223) tumors in terms of BMI in patients with endometrial cancers with early stage endometrioid-type adenocarcinoma histopathology [15]. Similarly, in our study, no difference was found according to wall involvement in tumors originating from the uterine corpus.

In studies comparing patients with endometrioid-type adenocarcinoma with and without LUSI in terms of myometrial invasion and grade, a notable difference was observed in regard to myometrial invasion. Still, no meaningful difference was found regarding grade [15,16]. In other studies, a notable difference was observed for both myometrial invasion and grade [14,17]. In our study, no meaningful difference was observed either in regard to myometrial invasion or grade according to the wall of the corpus from which the tumors originated. In addition, in a study by Erkaya et al., a significant difference was found for tumor size, while in our study, tumor size was determined not to be related to the wall from which the tumor originated [17].

Involvement of the lower uterine segment is linked to a higher likelihood of LVSI, even in the initial stages. Studies have found that LVSI is more prevalent in patients with lower uterine segment involvement [18,19,20]. Our study also found a significant difference in LVSI for corpus tumors with anterior and posterior wall involvement compared with tumors originating from the fundus (*p* = 0.038).

LUSI is recognized as a poor prognostic factor [7]. In some studies, it has been considered a risk factor for lymph node involvement, myometrial invasion, and lymphovascular invasion [20]. Studies comparing patient groups with and without LUSI in terms of prognosis observed a meaningful difference in favor of DFS and TS in the groups without LUSI in regard to overall and disease-free survival [7,19,21]. Another study found no significant difference in mortality and that lower segment involvement was not significant in terms of DFS and OS [17]. In another study, LUSI was not found to be associated with a high recurrence rate [21]. In our study, DFS and OS were not affected by wall involvement, but recurrence and ex rates were significantly higher in the case of anterior or posterior wall involvement compared with fundal involvement.

To our knowledge, this is the first study to investigate prognostic differences according to specific uterine wall locations in tumors confined to the corpus. The less aggressive course observed in fundal and lateral tumors may be related to anatomical factors, such as their greater distance from cervical and parametrial lymphovascular channels and a more limited lymphatic drainage network in these regions [22]. Furthermore, regional variations in endometrial gland density and hormonal exposure—particularly differences in estrogen and progesterone receptor distribution—may influence tumor biology and behavior [23]. These anatomical and hormonal characteristics could partly explain the lower LVSI rates and better clinical outcomes associated with fundal and lateral tumors.

In the present study, anterior and posterior tumors were significantly associated with higher rates of LVSI, recurrence, and mortality compared with fundal and lateral tumors. The observed odds of LVSI were approximately five times higher in anterior/posterior tumors, while recurrence and mortality were markedly elevated in these groups, with no events recorded in fundal and lateral tumors. In the Kaplan–Meier analysis, anterior and posterior tumors showed earlier recurrence and mortality events compared with fundal and lateral tumors; however, these differences did not reach statistical significance, likely due to the small number of events in the fundal and lateral groups and the limited sample size, which reduced the statistical power. Previous studies have demonstrated that tumor location within the uterus can influence patterns of lymphatic drainage and metastatic spread, with tumors on the anterior and posterior walls more frequently involving pelvic nodal basins and demonstrating aggressive pathological features [9,10,24]. Nevertheless, the visual separation of survival curves supports the hypothesis that primary tumor location within the uterine corpus may influence prognosis and that anterior/posterior involvement is associated with more aggressive clinical behavior. Our findings support these anatomical and biological considerations, suggesting that primary tumor site may serve as a prognostic indicator and should be taken into account in risk stratification and adjuvant treatment planning and that tumors originating from the anterior and posterior walls may require closer monitoring.

A key novelty of our work lies in evaluating the prognostic implications of tumor location within the uterine corpus, an aspect not previously analyzed in early stage endometrioid carcinoma. This focus may contribute to refining risk stratification models and tailoring follow-up protocols. The relatively small sample size—particularly the lateral wall subgroup (*n* = 4)—reduced the statistical power and increased the likelihood of a Type II error, potentially obscuring true differences in survival [25]. Future research directions should include prospective multicenter cohort studies to increase statistical power, the incorporation of molecular profiling to investigate tumor heterogeneity, and the use of advanced functional imaging modalities—such as dynamic contrast-enhanced MRI—that capture regional microvascular and physiological differences and may help clarify tumor behavior based on location.

One of the most significant advances in the diagnosis and treatment of endometrial carcinomas over the past decade has been the ability to molecularly distinguish and classify these carcinomas. Molecular classification has also been introduced in the FIGO 2023 staging system for endometrial cancer [26]. Molecular characteristics can be used to predict the risk of recurrence and, consequently, survival [27,28]. The most impactful molecular classification is that proposed by The Cancer Genome Atlas (TCGA), which classifies endometrial carcinomas into four categories: (1) POLE Mutant (POLE mut) (good prognosis); (2) microsatellite instability—high/hypermutated (MSI-high)/Mismatch Repair (MMRd); (3) p53 Anormal (p53 abn) (poor prognosis); and (4) Non-Specific Molecular Profile (NSMP) [29]. The finding that the prognosis was worse in tumors originating from the anterior and posterior walls in our study could perhaps be explained by the molecular characteristics of the tumor; however, due to the retrospective nature of our study and the lack of widespread molecular studies, we were unable to make this assessment. Therefore, we have no data to suggest that a mutation with a poor prognosis (p53abn mutation) may be present in tumors originating from the anterior and posterior walls from a molecular perspective. Speculatively, it might be appropriate to perform a p53abn mutation study in tumors originating from the anterior and posterior walls.

The limitations of this study include its retrospective nature, small number of cases, and lack of molecular classification. Despite these limitations, its strength lies in being the first study to assess the prognostic impact of tumor location within the corpus in early stage endometrioid carcinoma.

## 5. Conclusions

This study determined that LVSI and ex rate are higher in patients with tumors originating from the anterior and posterior walls. The high ex rate due to these two wall localizations may be due to LVSI, which is another prognostic factor. We believe that the prognosis may be poor for UC tumors originating from the anterior and posterior walls. Closer follow-up may be necessary for endometrial cancers originating from the anterior and posterior uterine walls. However, further prospective studies with more cases are required.

## Figures and Tables

**Table 1:** Patient demographic, pathology, and survival data.

Features	Patients (*n* = 68)
Age	58.5 (54–67.75)
BMI (kg/m^2^)	36 (30.8–43.8)
Nulliparity	1 (1.5)
DM	15 (22.1)
HT	19 (27.9)
Grade 1	17 (25)
Grade 2	47 (69.1)
Grade 3	4 (5.9)
Tumor Size (mm)	40 (30–50)
Stage 1A	51 (75)
Stage 1B	17 (25)
Anterior Wall	13 (19.1)
Posterior Wall	10 (14.7)
Fundus	41 (60.3)
Lateral Wall	4 (5.9)
LVSI	16 (23.5)
Recurrence	8 (11.8)
DFS (month)	67 (60–74)
OS (month)	67 (61–74.75)
Ex	8 (11.8)
Alive	60 (88.2)

BMI, body mass index; DM, diabetes mellitus; HT, hypertension, LVSI, lymphovascular space invasion; DFS, disease-free survival; OS, overall survival. Age, BMI, tumor size, DFS, and OS are given as means (quartiles); other data are given as *n* (%).

**Table 2:** Comparison of demographic, pathology, and survival results according to the region of the uterine wall in which the tumor originated.

Features	Anterior (*n* = 13)	Posterior (*n* = 10)	Fundus (*n* = 41)	Lateral (*n* = 4)	*p* Value
Age	58 (53–67)	60 (57–63)	56 (52–63)	64 (57–68)	0.388
BMI (kg/m^2^)	37.5 (36–44)	34.2 (30–44)	33 (27–44)	36 (36–36)	0.520
Nulliparity	1 (7.5)	0 (0)	0 (0)	0 (0)	0.231
DM	2 (15.4)	4 (40)	8 (19.5)	1 (25)	0.497
HT	2 (15.4)	4 (40)	12 (29.3)	1 (25)	0.616
Grade 1	3 (23.1)	1 (10)	12 (29.3)	1 (25)	0.501
Grade 2	9 (69.2)	9 (90)	27 (65.9)	2 (50)	0.501
Grade 3	1 (7.7)	0 (0)	2 (4.9)	1 (25)	0.501
Tumor Size (mm)	40 (32–47)	45 (32–52)	35 (26–50)	20 (12–42)	0.320
Stage 1A	8 (61.5)	8 (80)	32 (78)	3 (75)	0.661
Stage 1B	5(38.5)	2 (20)	9 (22)	1 (25)	0.661
LVSI	5 (38.5)	5 (50)	5 (12.2)	1 (25)	**0.038**
Recurrence	4 (30.8)	4 (40)	0 (0)	0 (0)	**<0.001**
DFS (month)	68 (63–71)	64.5 (42–67)	67 (60–75)	65 (61–72)	0.310
OS (month)	68 (66–75)	66.5 (52–70)	67 (60–75)	65 (61–72)	0.632
Ex	4 (30.8)	4 (40)	0 (0)	0 (0)	**<0.001**
Alive	9 (69.2)	6 (60)	41 (100)	4 (100)	**<0.001**

BMI, body mass index; DM, diabetes mellitus; HT, hypertension, LVSI, lymphovascular space invasion; DFS, disease-free survival; OS, overall survival. Age, BMI, tumor size, DFS, and OS are given as means (quartiles); other data are given as *n* (%).

**Table 3:** Association between tumor location and LVSI, recurrence, and mortality with odds ratios and 95% confidence intervals.

Outcome	Anterior/Posterior *n*/N (%)	Fundus/Lateral *n*/N (%)	OR (95% CI)	*p* Value
LVSI	10/23 (43.5%)	6/45 (13.3%)	5.00 (1.52–16.45)	**0.013**
Recurrence	8/23 (34.8%)	0/45 (0.0%)	48.00 (2.60–886.66)	**<0.0001**
Mortality	8/23 (34.8%)	0/45 (0.0%)	48.00 (2.60–886.66)	**<0.0001**

LVSI, lymphovascular space invasion; OR, odds ratio; CI, confidence interval.

## Data Availability

The data that support the findings of this study are available from the corresponding author upon reasonable request.
